# Generation of a Novel Bacteriophage Library Displaying scFv Antibody Fragments from the Natural Buffalo Host to Identify Antigens from Adult *Schistosoma japonicum* for Diagnostic Development

**DOI:** 10.1371/journal.pntd.0004280

**Published:** 2015-12-18

**Authors:** Christopher G. Hosking, Hamish E. G. McWilliam, Patrick Driguez, David Piedrafita, Yuesheng Li, Donald P. McManus, Leodevico L. Ilag, Els N. T. Meeusen, Michael J. de Veer

**Affiliations:** 1 Department of Physiology, Monash University, Clayton, Victoria, Australia; 2 Department of Microbiology and Immunology, The University of Melbourne, the Peter Doherty Institute for Infection and Immunity, Parkville, Victoria, Australia; 3 QIMR Berghofer Medical Research Institute, Brisbane, Queensland, Australia; 4 School of Applied Sciences and Engineering, Federation University, Churchill, Victoria, Australia; 5 Bio21 Molecular Sciences and Biotechnology Institute, University of Melbourne, Parkville, Victoria, Australia; 6 Department of Microbiology, Monash University, Clayton, Victoria, Australia; McGill University, CANADA

## Abstract

The development of effective diagnostic tools will be essential in the continuing fight to reduce schistosome infection; however, the diagnostic tests available to date are generally laborious and difficult to implement in current parasite control strategies. We generated a series of single-chain antibody Fv domain (scFv) phage display libraries from the portal lymph node of field exposed water buffaloes, *Bubalus bubalis*, 11–12 days post challenge with *Schistosoma japonicum* cercariae. The selected scFv-phages showed clear enrichment towards adult schistosomes and excretory-secretory (ES) proteins by immunofluorescence, ELISA and western blot analysis. The enriched libraries were used to probe a schistosome specific protein microarray resulting in the recognition of a number of proteins, five of which were specific to schistosomes, with RNA expression predominantly in the adult life-stage based on interrogation of schistosome expressed sequence tags (EST). As the libraries were enriched by panning against ES products, these antigens may be excreted or secreted into the host vasculature and hence may make good targets for a diagnostic assay. Further selection of the scFv library against infected mouse sera identified five soluble scFv clones that could selectively recognise soluble whole adult preparations (SWAP) relative to an irrelevant protein control (ovalbumin). Furthermore, two of the identified scFv clones also selectively recognised SWAP proteins when spiked into naïve mouse sera. These host B-cell derived scFvs that specifically bind to schistosome protein preparations will be valuable reagents for further development of a cost effective point-of-care diagnostic test.

## Author Summary

Mass drug administration using the highly effective drug praziquantel (PZQ) is currently the method of choice to combat schistosomiasis. However, this treatment regime has limitations; in particular, it does not prevent re-infection and sporadic parasite resistance against PZQ is a continuing threat. The path to the successful control of schistosomiasis is highly challenging and must consider, not only the complex nature of the host-parasite interaction, but also the capacity to assess disease burden and parasite re-emergence in communities where successful control has been achieved. Furthermore, control programs must be economically sustainable in endemic countries and despite significant recent advancements the elimination of schistosomiasis may still be some time away. Accordingly, there is a definitive need to formulate innovative approaches for the development of improved diagnostic tools to accurately assess the disease burden associated with active schistosome infections. Here we describe the usefulness of a phage display library to mature antibody fragments derived from lymph node RNA of the natural buffalo host of the Asian schistosome, *Schistosoma japonicum*, following an experimental infection. These mature antibody fragments were able to bind native parasite proteins and could thus be used to develop a low cost and accurate point-of-care diagnostic test.

## Introduction

Schistosomiasis is one of the most insidious of all the tropical parasitic infections and threatens the health of hundreds of millions of people worldwide [1]. The last 20 years has seen remarkable progress in disease control through the use of praziquantel (PZQ), but this drug does not protect against re-infection and mass drug administration programmes based around its use are probably untenable long term [2–4]. Recently there has been a major focus on the development of anti-schistosome vaccines, but to date a protective commercial vaccine remains elusive [1]. As mass drug administration decreases worm burdens within endemic areas, the need for improved diagnostic tests should be given research priority [5]. However, in countries where elimination of schistosomiasis has been given precedence, case detection of infected individuals remains problematic as the commonly used methods for diagnosis lack the necessary sensitivity and specificity to accurately determine parasite burden [6]. Although application of modern research laboratory techniques has seen improvements in the diagnosis of helminth infection, uptake has not been uniform and proof of concept studies that show promise have often not been followed through with much needed product development [7].

Currently the Kato-Katz thick smear stool method, based on detection of eggs in faeces, is the test sanctioned by the World Health Organization (WHO) for qualitative and quantitative diagnosis of intestinal schistosomiasis [8, 9]. This test is generally specific, simple and relatively inexpensive, but like many parasitological tests, sensitivity can be insufficient, particularly when worm burdens are low [7]. Consequently, the use of single Kato-Katz measurements can underestimate the prevalence of infection and can confound confirmation of cure assessment following chemotherapy [10]. This is of particular importance in the Peoples Republic (PR) of China as the country moves towards programs aimed at the elimination of schistosomiasis japonica [11, 12]. Since the 1950’s the prevalence of schistosomiasis japonica within Chinese provinces has dramatically decreased [13, 14] and the requirements for a diagnostic tool has moved from the detection of parasitic infection to the ability to effectively assess disease prevalence [14].

Evaluation of the Gates-funded SCORE project in African countries demonstrated that a rapid, accurate point-of-care (POC) diagnostic test that detects a circulating cathodic antigen (CCA) could identify *S*. *mansoni* antigens [15, 16]. The CCA and circulating anodic antigen (CAA) have been investigated as potential diagnostic candidates and can be detected in the serum and urine of infected individuals [17, 18]. These antigens are cleared from the serum and urine of schistosomiasis patients within weeks following curative treatment [19]. However, success of these tests has only been validated for areas of high and moderate endemicity [18, 20]. Whilst CCA and CAA appear to be excellent antigen based tests, we have taken a different approach that may offer advantages for the development of reagents aimed at detecting very low infection levels.

Recently McWilliam et al., demonstrated, in a rat model of schistosomiasis, that the developing schistosome worm can elicit a distinct immune response in discrete tissue sites [21]. Building on this concept we previously published the construction of an scFv-phage library for the detection of larval stage antigens as potential vaccine candidates [22]. However, the *S*. *japonicum* larval stages are small, transient and rapidly migrate between tissues. The adult parasites are much larger, more persistent and shed antigen directly into the blood which makes them much more attractive targets for an antigen based diagnostic. Here we describe the construction and characterisation of scFv libraries derived from the portal lymph nodes of *S*. *japonicum* infected *B*. *bubalis*, and demonstrate their ability to bind to the surface of adult *S*. *japonicum* worms and excretory-secretory (ES) products. These reagents offer many advantages for diagnostic development, including the ability to affinity mature the reagents, easy selection in a number of modalities, existing detection reagents and strong binding. It is hoped these reagents can be developed into a rapid POC diagnostic to aid in the surveillance and eventual elimination of *S*. *japonicum*.

## Materials and Methods

### Ethics statement

Written approval for animal experiments was provided by the Ethical Review Board of the Hunan Institute of Parasitic Diseases (approval # 110818) and from Monash University Animal Ethics Committee (approval # 2011-124-FW). Animals were maintained and cared for according to the Animal Ethics and Procedures Guidelines of the PR China.

### Parasite collection and crude protein extracts

Cercariae from *S*. *japonicum* were shed from infected *Oncomelania hupensis* snails collected from an endemic region in the People’s Republic (PR) of China using described methods [23, 24]. Adult *S*. *japonicum* worm pairs were collected from infected mice at QIMR Berghofer Institute for Medical Research as described [25]. Extracts of soluble whole adult preparations (SWAP) or excretory-secretory (ES) products from live adult worms were prepared as described [26, 27]. Soluble egg antigen (SEA) was generated from eggs extracted from infected livers digested in 400 μg/ml collagenase B overnight at 37°C. The digested liver solution was centrifuged (400 *g*, 5 min) and washed extensively in PBS. The washed solution was then passed though sieves (250μm and 150μm, respectively) and eggs were separated out of the resulting solution using a Percoll gradient and exposed to 10 freeze thaw cycles. Eggs were then homogenised and centrifuged at 10,000 *g* for 2 hr at 4°C and the soluble fraction (SEA) collected. Protein concentrations were determined by bicinchoninic acid assay (BCA; Thermo Fisher Scientific, USA).

### Experimental animal infections and sample collection

Animal experiments and sample collection were conducted in the PR China as described [28]. Briefly, six mixed sex *S*. *japonicum* infected *B*. *bubalis* were obtained from an *S*. *japonicum*-endemic region in Hunan Province, PR China. Pre-existing infection status was confirmed by faecal egg count to provide animals where natural immunity could be “boosted” by subsequent experimental schistosome infection. All animals were drenched upon arrival with PZQ (25 mg/kg) and randomly assigned into two experimental groups (*n* = 3 per group). Group 1 was infected with 400 live *S*. *japonicum* cercariae percutaneously on the inner thigh. Group 2 was the uninfected control group. Animals were sacrificed 11–12 days post infection (days p.i.).

### Collection of liver-draining lymph node samples

Lymph nodes draining the liver of *B*. *bubalis* were collected and cells isolated as described previously [29]. For RNA preparation, 1 x 10^9^ cells were centrifuged and the cell pellet resuspended in 2 ml of QIAzol lysis buffer (QIAGEN, Netherlands) and RNA was prepared according to the manufacturer’s recommendations. RNA was further purified using an RNeasy Mini Kit (QIAGEN, Netherlands) as per the manufacturer’s recommendations. RNA was quantitated via absorbance at 260 nm using a NanoDrop spectrophotometer (Thermo Fisher Scientific, USA) and stored at -80°C until required.

### Construction and amplification of B-cell scFv-phage antibody libraries

The preparation and characterisation of a scFv-phage display library similar to that used in this study has been described previously [22]. Briefly, full length variable light (VL) and variable heavy (VH) chain genes were amplified by PCR from portal-lymph node RNA derived from buffalo 11–12 days following an experimental *S*. *japonicum* infection. The scFv fragment was cloned into a phage display vector (pAK100; Plückthan Laboratory, University of Zurich, Switzerland) with the VL and VH genes separated by DNA encoding a flexible linker sequence (VL-(G_4_S)_4_-VH). The scFv fragment was directionally cloned into the pAK100 vector using differential *Sfi*I restriction sites and was fused in-frame to the phage gene III coding DNA. This library was transformed in XL-1 Blue *Escherichia coli* cells and approximately 5 x 10^7^ transformants were recovered following overnight incubation at 37°C. The panning library was amplified by inoculating 50 ml of non-expressing (NE) medium (2 X Yeast Tryptone (2YT), 1% glucose, 25 mg/ml chloramphenicol) with approximately 10^9^ XL-1 Blue *E*. *coli* cells containing scFv phagemids and shaken at 37°C. At OD_600_ = 0.5, 1 x 10^11^ transducing units per ml (TU/ml) M13K07 helper-phage (New England Biolabs, USA) and 25 μl 1 M Isopropyl β-D-1-thiogalactopyranoside (IPTG; Sigma-Aldrich, USA) solution were added and culture incubated for 15 mins at 37°C without agitation. The culture was then centrifuged at 3500 *g* for 10 mins and the resulting pellet was diluted in 100 ml low-expression (LE) medium (2YT, 1% glucose, 25 mg/ml chloramphenicol (Sigma Aldrich, USA) and 0.5 mM IPTG) and shaken for 16 hr at 37°C for phage production. Two hours post infection, 30 mg/ml kanamycin (Bioline, Australia) was added. Resulting scFv-phage particles were purified and concentrated 100-fold by PEG NaCl precipitation [30], resuspended in phosphate buffered saline (PBS), and stored at 4°C. Following overnight culture, phage titres of 1 x 10^11^–1 x10^12^ TU/ml were typically observed. Plasmid DNA was sequenced by the Micromon Sequencing Facility (Monash University, Australia) and aligned to *Bos taurus* antibody protein sequences using Clustal Omega Multiple Sequence Alignment Software [31].

### Selection of scFv-phage binders to adult worms or excretory-secretory products

For selection of high affinity binders, 1 x 10^11^ scFv-phage particles in 1ml PBS were initially pre-absorbed in 1.7 ml microfuge tubes (Axygen, Corning Life Sciences, USA) or 96 well microplates (Corning Life Sciences, USA) for 1 hr at RT with agitation. This was repeated a total of four times to eliminate scFv-phages that preferentially bind plastic. Following pre-absorption, 1 x 10^11^ TU/ml scFv-phages were added to 10 ± 2 formaldehyde-fixed adult schistosome pairs (10% formaldehyde for 30 mins followed by 3 washes with PBS) or 1 μg of ES worm products (0.1 μg coated per well on a on 96 well plate) and allowed to bind for 2 hr at RT with gentle agitation. Control reactions of scFv-phages without the addition of parasite material were also prepared. Tubes and microplates were then washed 10 times with PBS with 0.05% (v/v) Tween-20 (PBS-T), followed by an additional two washes with PBS. Bound scFv-phages were eluted with 0.2 M glycine/HCl, pH 3.0 for 15 min at RT. Supernatant from tubes and microplates was then collected and immediately neutralised with appropriate volume of 1 M Tris-HCL. Eluted scFv-phages (typically 1 x 10^4^–1 x10^6^ TU/ml) were amplified as previously outlined and resuspended in PBS. Amplified scFv-phages were used for further panning rounds or parasite binding analysis. Libraries panned against adults or ES are termed Bp-R3-A or Bp-R3-ES respectively and an equal mix of each scFv-pool is termed Bp-R3-AES. Combined BP-R3-AES phage pools were used to minimise amount of *S*. *japonicum* material required.

### Selection against infected mouse sera

Naïve and *S*. *japonicum* infected mouse sera (21 days p.i.; kindly supplied by Dr. Patrick Driguez, QIMR Berghofer Medical Research Institute, Australia) were treated with Affi-Gel Blue to remove albumin as per the manufacturer’s instructions (Bio-Rad, USA). The Bp-R3-AES phage pool was adjusted to 1 x 10^11^ TU/ml and absorbed against depleted naïve mouse sera (diluted 1:10 in PBS) coated onto 96 well microtitre plates (100 μl/well; Maxisorb; NUNC, Denmark). Pre-absorbed Bp-R3-AES were then panned against depleted infected mouse sera (diluted 1:10 in PBS) coated onto 96 well microtitre plates (100 μl/well; Maxisorb; NUNC, Denmark). Following each panning round phages were eluted and amplified as previously outlined and again absorbed against depleted naïve mouse sera, before being further panned against depleted infected mouse sera. This process was repeated for a total of three rounds. This post infected mouse sera panned library will be termed Bp-R3-post infected mouse sera (Bp-R3-PIMS).

### Generation of soluble scFv clones

Selected scFv coding regions from Bp-R3 phages following infected mouse sera panning (Bp-R3-PIMS) were sub-cloned into the *E*. *coli* expression vector pAK600 (Plückthun Laboratory, University of Zurich, Switzerland) and transformed using electroporation into TOP 10 F’ *E*. *coli* cells (Life Technologies, USA) as previously described [32]. The recombinant scFv are expressed with an alkaline phosphatase (AP) tag to aid in solubility and to facilitate dimerisation and direct detection [33]. The scFv-AP fusions were purified by ion exchange chromatography (HiTrap Q FF, GE Healthcare, UK) according to the manufacturer’s recommendations. Purity and correct size of scFv-AP fusion proteins were assessed by western blot. Soluble scFv clones were sequenced by the Micromon Sequencing Facility (Monash University, Australia) and aligned using Clustal Omega Multiple Sequence Alignment Software [31]. Soluble scFv fusion proteins were designated Bp-scFv-1 to Bp-scFv-5, respectively.

### Binding of Bp-R3 scFv-phages and soluble scFvs clones to schistosome extracts by ELISA

Microplates (Corning Life Sciences, USA) were coated with 100 μl of either *S*. *japonicum* SWAP, ES, SEA, ovalbumin (0.5 μg/well) or SWAP spiked into uninfected mouse sera (equivalent to 0.5 μg/well in 1:10 diluted naïve mouse sera) in carbonate coating buffer (0.05 M carbonate-bicarbonate, pH 9.6) and incubated overnight at 4°C. The next day microplates were washed three times and blocked in PBS-T for 1 hr at 37°C. The scFv-phage pools (Bp-pre, Bp-R3-A, Bp-R3-ES, Bp-R3-AES or Bp-R3-PIMS) diluted 1:5 in PBS-T and soluble scFv-AP clones diluted 1:10 in PBS-T were added to triplicate wells and incubated for 1 hr at 37°C. Specific binding of scFv-phage pools was detected using an anti-M13 pIII monoclonal antibody (New England Biolabs, USA) followed by biotin-conjugated anti-mouse antibody (goat anti-mouse IgG Fc, Jackson Immunoresearch), then streptavidin-HRP (BioRad, USA). Reactivity of scFv-AP clones towards schistosome antigens was detected using rabbit anti-alkaline phosphatase (ABCAM, USA), then swine anti-rabbit-HRP conjugate (Dako, Germany) All detection antibodies were diluted 1:1000 in PBS-T and incubated for 1 hr at 37°C. Following incubation, all plates were washed three times with PBS-T and developed with 3,3’,5,5’-tetramethylbenzidine (TMB) solution (Life Technologies, USA) for 15 minutes and the reaction was stopped with 2 M H_2_SO_4_. Antibody or scFv-phage binding was detected using O.D. measurements at 450 nm.

### Western blotting

Protein preparations (SWAP or SEA) were resolved by reducing SDS-PAGE and transferred to nitrocellulose membranes as per the manufactures recommendations (iBlot Dry Blotting System; Life Technologies, USA). Membranes were visualised with Ponceau S (0.1% (w/v) Ponceau S in 5% acetic acid; Sigma-Aldrich, USA), cut into individual lanes and blocked for 2 hr at RT in PBS-T. Membranes were incubated overnight at 4°C with Bp-R3-AES phages (diluted 1:5 in PBS-T). Individual lanes were washed three times with PBS-T and Bp-R3-AES phages were detected with anti-M13 pIII monoclonal antibody (New England Biolabs, USA), followed by biotin-conjugated anti-mouse antibody (goat anti-mouse IgG Fc, Jackson Immunoresearch, USA), then streptavidin-HRP (BioRad, USA). All detection antibodies were diluted 1:1000 in PBS-T and incubated for 1 hr at RT. Protein bands were detected using the metal enhanced DAB peroxidase substrate detection system (Thermo Fisher Scientific, USA).

### Carboxyfluorescein succinimidyl ester (CFSE) conjugation and fluorescent microscopy

Discrete amplified scFv-phages (Bp-pre, Bp-R3-A or Bp-R3-ES) were diluted to 1 x 10^11^ TU/ml and labelled with 120 μg of carboxyfluorescein succinimidyl ester (CFSE; 10 mg/ml in DMSO) in a total volume of 1 ml PBS for 1 hr at RT in the dark, with agitation. Excess CFSE was removed by overnight dialysis against PBS at 4°C. Microscopy was conducted using CFSE labelled scFv-phages (Bp-pre, Bp-R3-A or Bp-R3-ES). Briefly, 10 ± 2 formaldehyde-fixed adult worm pairs were reacted with 1 x 10^9^ TU/ml phage particles diluted in PBS for 1 hr at RT in the dark. Parasites were washed five times with 1.5 ml of PBS-T and an additional two times with PBS. Parasites were mounted on slides using PBS with 10% glycerol (Merck-Millipore, Germany). Images were taken using a Zeiss fluorescence microscope (Zeiss Group, Germany) and a 2.5x objective with a consistent exposure time for each sample. Images were processed using Zen software (Zeiss Group, Germany). At least 10 adult parasites were observed for each represented image. Confocal microscopy was performed using a Nikon SMZ 25 stereomicroscope with a motorised stage with CFSE labelled Bp-R3-A phage and formaldehyde-fixed adult parasites. Two hundred images were captured at 10.8 μm steps with fixed exposure times and an SHR Plan Apo 2x objective. The images shown were reconstructed and rendered using ImageJ software [34].

### Protein microarray screening and scanning

A microarray consisting of 232 schistosome specific proteins was prepared as described [35]. Briefly, array pads were hydrated with Whatman Blocking Buffer (WBB; Whatman, GE Healthcare, UK). Phages (M13K07 helper-phage control, Bp-R3-A or Bp-R3-ES phages) were pre-absorbed in WBB containing 10% (w/v) reconstituted *E*. *coli* lysate (EBB; Mc Lab, USA). Binding of scFv-phage particles was detected by addition of anti-M13 pIII monoclonal antibody (diluted 1:1000 in WBB; New England Biolabs, USA), biotin-anti-mouse antibody (diluted 1:1000 in WBB; Jackson Immunoresearch, USA), then streptavidin-conjugated Cy5 fluorophore (diluted 1:200 in WBB; Surelight P3, Columbia Biosciences, USA). Microarray pads were washed 3 times with TBS-T, 3 times with TBS, then in ultrapure water and dried by centrifugation at RT in a 50 ml Falcon tube (BD Biosciences, USA) for 5 min at 500 *g*.

Microarray slides were scanned using a confocal laser microarray scanner (Genepix 4300A, Molecular Devices, USA). The signals were quantified with image analysis software (Genepix Pro 7, Molecular Devices, USA) and the reported feature intensity was calculated by subtracting the median local background signal from the feature signal.Data was analysed using a variation of the “group average” method [36, 37]. Signal intensity (S.I.) greater than the average of the “No DNA” negative controls plus 2.5 standard deviations (S.D.) and no binding by M13K07 helper-phage was used to determine positive recognition. Binding intensity was designated on a plus minus scale. Sequences of identified antigens were then analysed for developmental expression based on previous work [38] and EST Profile Viewer (http://www.ncbi.nlm.nih.gov).

### Statistical analysis

Differences between scFv-phages or soluble scFv clones was determined using a one- or two-way analysis of variance test (ANOVA) followed by a Tukey’s post-hoc test. Statistical results were reported when significance was achieved at *P* < 0.05. Statistical analysis was performed using GraphPad Prism, version 6.01 software.

## Results

### Generation and enrichment of a scFv-phage library from infected buffalo portal-lymph nodes

The VH and VL antibody regions were amplified from buffalo portal lymph nodes 11–12 days post infection with *S*. *japonicum* and cloned into a scFv-phage vector (S1A Fig). Sequencing of assembled scFv fragments revealed framework similarities and distinct variation in the complementarity determining regions (CDR; S1B Fig). Transformation into XL-1 Blue *E*. *coli* cells produced approximately 1.5 x 10^7^ colonies. Panning against whole fixed adult worm pairs and ES products resulted in relative enrichment levels of greater than 800 or 80 fold, respectively, (S2 Fig). Libraries generated from this material are prefixed with Bp-R3.

### Bp-R3 scFv-phage libraries bind to adult schistosomes and ES products

**Fig 1:**
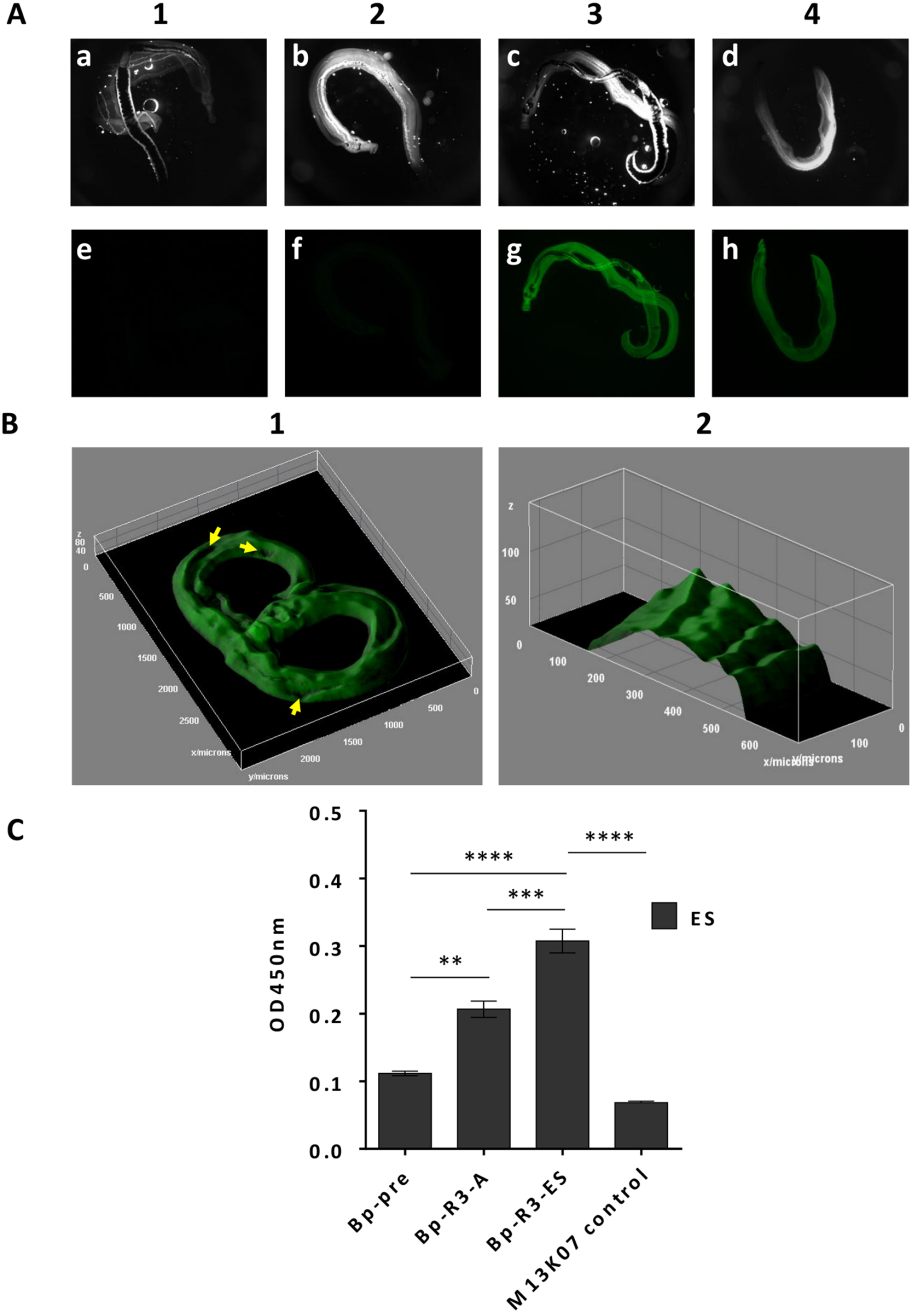
Buffalo portal-round three selected (Bp-R3) single-chain Fv domain (scFv) phage recognise adult *Schistosoma japonicum* parasites and extracts. Identical numbers of carboxyfluorescein succinimidyl ester (CFSE) conjugated non-scFv expressing M13K07 helper-phage (A1), Bp-pre (A2), Bp-R3-A (A3) or Bp-R3-ES (A4) scFv-phage pools were incubated with formaldehyde-fixed adult worm pairs. Whole *S*. *japonicum* adult parasite images captured with consistent exposure times are presented as dark field (A1, a-d) and FITC fluorescence images (A1, e-h). Three dimensional rendered image of CFSE labelled Bp-R3-AES scFv-phages binding to adult *S*. *japonicum* (B1). A total of two hundred images were captured at 10.8 μm steps with consistent exposure time. Arrows represent areas of minimal binding (B1). Cross sectional view of scFv-phage binding to adult schistosome showing binding is restricted to the surface of the parasite, with no observable binding to internal structures (B2). Bp-R3 scFv-phages bind to *S*. *japonicum* excretory-secretory (ES) protein extracts by ELISA (C). Data represent mean ± S.E.M. of the O.D._450nm_ for discrete scFv-phage pools binding to ES protein preparations (C). Statistical significance was determined by one-way ANOVA followed by Tukey’s post-hoc test, where ** *P* < 0.01, *** *P* < 0.001, **** *P* < 0.0001.

**Fig 2:**
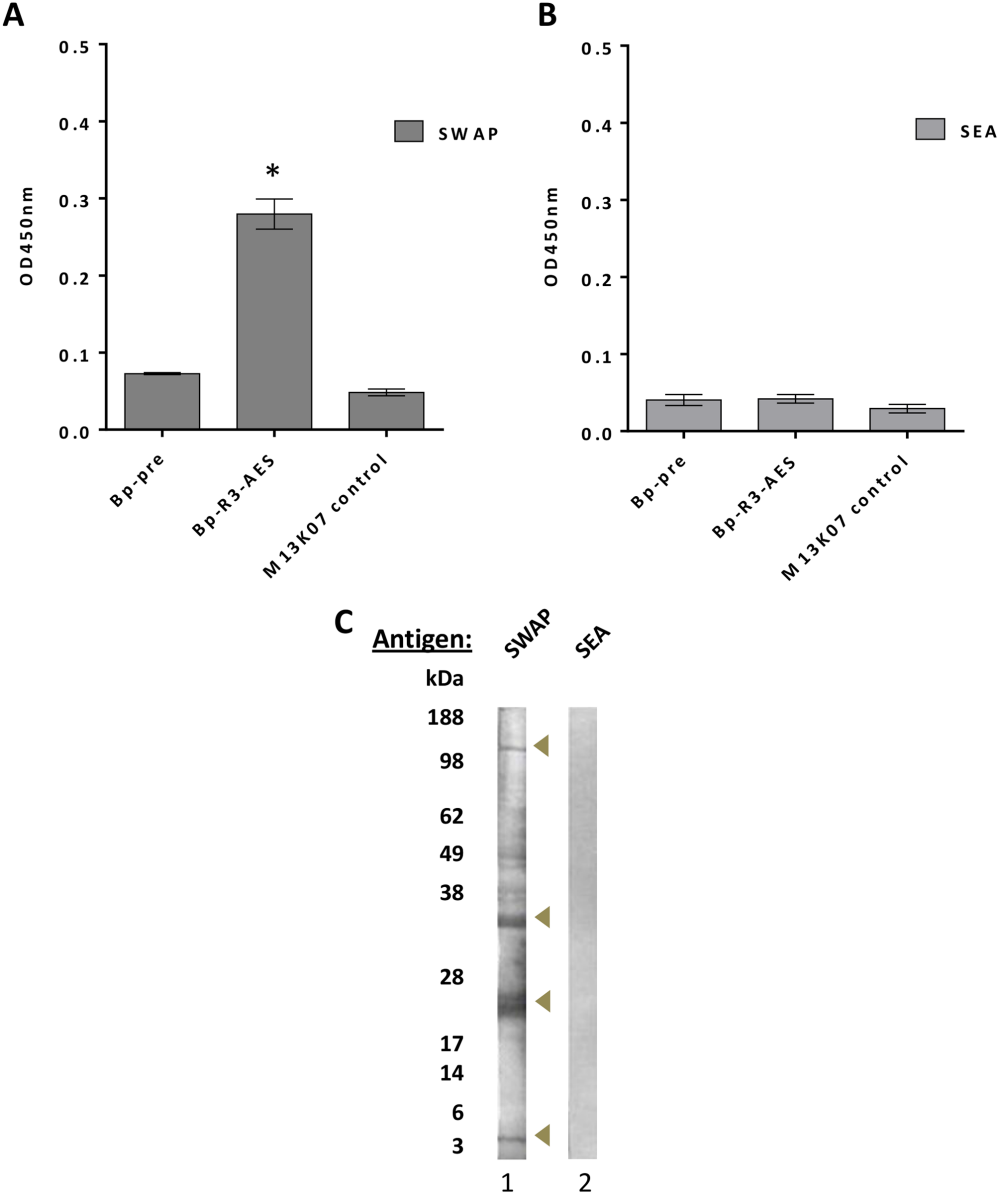
Binding of buffalo portal-round three selected-adult and ES pooled (Bp-R3-AES) single-chain Fv domain (scFv) phage library to *Schistosoma japonicum* protein extracts. Data represent mean ± S.E.M. of the O.D._450nm_ for Bp-R3-AES phage binding to soluble whole adult preparations (SWAP) (A) and soluble egg antigens (SEA) (B). Statistical significance was determined by one-way ANOVA followed by Tukey’s post-hoc test, where * *P* < 0.05. Antigens from SWAP (Lane 1), and SEA (Lane 2) of *S*. *japonicum* were probed with Bp-R3-AES phage (C). Molecular weights in kilodaltons (kDa) are indicated on the left hand side. Regions of intense binding by Bp-R3 scFv phages to antigen preparations is indicated by arrows.

Bp-R3 phages were mixed with formaldehyde-fixed adult worm pairs and binding was assessed by fluorescent staining (Fig 1). Following three rounds of panning against intact adult worms CFSE labelled Bp-R3-A and Bp-R3-ES phage libraries displayed increased fluorescent binding to adult worm pairs under constant exposure conditions compared with CFSE labelled phage prior to selection (Bp-pre) and control M13K07 helper phage (Fig 1A 3–4 compared to 1–2). To determine where on the adult schistosome the Bp-R3 phage bound we performed confocal microscopy using CFSE labelled Bp-R3 phages and adult schistosomes (Fig 1B). It demonstrated that binding to fixed parasites was primarily restricted to the surface, with no apparent binding to internal structures (Fig 1B-2). Interestingly, although initially binding appeared to be uniform (Fig 1A), confocal examination indicated there are regions on the surface of the parasite where phage do not bind in sufficient numbers to obtain a positive fluoresce signal (Fig 1B-1 arrows). To quantitate phage binding to schistosome ES products we coated an ELISA plate with ES antigens and added different phage libraries, this showed that the enriched Bp-R3-ES phage library displayed significantly greater binding to ES products than any other phage library (Fig 1C). The Bp-R3-A library selected for adult binding also bound ES antigens more strongly than control phage, indicating the ES and adult surface likely share cross-reactive epitopes (Fig 1C).

### Bp-R3 scFv-phage libraries recognise stage-specific antigens on protein microarrays

Screening of a schistosome specific protein microarray with Bp-R3 phages (Adult or ES) resulted in significant recognition of ten antigens. All ten of these antigens were recognised by the Bp-R3-ES phages, however only three antigens were consistently recognised by both the Bp-R3-A and Bp-R3-ES phages. These were a hypothetical protein (NCBI GenBank accession number **AY808393**), a protein similar to the myosin heavy chain (SJCHGC09420; **AY8153690**) and the previously defined Sjp40 protein (**AY814158**) (Table 1).

**Table 1:** *Schistosoma japonicum* protein microarray antigen recognition by buffalo portal-round three selected (Bp-R3) single-chain antibody Fv domain (scFv) phages.

		Recognition by R3-scFv-phage ^a^		Life cycle stage ^b^		
Protein name	NCBI GenBank Accession number	R3-scFv-A	R3-scFv-ES	EST	Protein	Schistosome specific ^c^
**SJCHGC02287; Tropomyosin**	**AY809972**	-	+++	A	CSAEM	No
**Hypothetical protein containing DNA-binding SAP domain**	**AY811797**	-	++	A	S	Yes
**Hypothetical protein**	**AY808393**	++	++++	A	S	Yes
**Calponin-like**	**AY813467**	-	+++	SA	CSA	No
**Hypothetical protein**	**AY814150**	-	++	SA	S	Yes
**Hypothetical protein**	**AY815838**	-	+	S	CA	Yes
**Sjp40; similar to P40_SCHMA Major egg antigen (P40)**	**AY814158**	++++	++++	SAEM	CSAEM	No
**SJCHGC05640; Putative dynein light chain-1**	**AY915388**	-	+	SAE	CSE	No
**Hypothetical protein**	**AY808749**	-	+	A	-	Yes
**SJCHGC09420; similar to myosin heavy chain**	**AY815690**	+++	++++	SA	CSAEM	No

^a^ Recognition level score: the average signal intensity of binding which was greater than 2.5 standard deviations of controls. Very high (++++) >1000 signal intensity (SI), High (+++) 500–999 SI; Moderate (++) 100–499 SI; low (+) 0–99 SI; (-) not a significant hit.

^b^ Life stage with highest expression is based on previous work [38]: cercariae (C), schistosomula (S), adult (A), egg (E) or miracidium (M).

^c^ Schistosome specific proteins are defined as those with no orthologues protein in other species via proteomic analysis [38].

Other antigens recognised by the Bp-R3 phages included an additional four hypothetical proteins (**AY811797**, **AY814150**, **AY815838** and **AY808749**) and a further four known proteins, including tropomyosin (**AY809972**), a calponin-like protein (**AY813467**), a putative dynein light chain-1 protein (DLC1; **AY915388**) and a protein similar to myosin heavy chain (**AY815690**). The tropomyosin antigen (SJCHGC02287) is a 25 kDa protein expressed in a range of tissues at different developmental stages and is associated with muscle contraction and cystoskeletal structure and function [39]. Although tropomysins are not specific to schistosomes, the SJCHGC02287 tropomyosin variant shares less than 50% homology with any other organism outside the schistosome genus. The calponin-like antigen shares homology with a 38 kDa *S*. *japonicum* calponin, which has been previously investigated [40]. The DLC1 from *S*. *japonicum* has previously been investigated as a potential target for vaccine or drug development [41]. The function of the **AY815838** hypothetical protein is currently unknown, but the sequence has some homology to the *S*. *mansoni* surface antigens Sm13 (**AAC25419.1**; 30%) and Sm25 (**AAA29943**; 34%). Interestingly, all of the schistosome specific antigens identified using the array show increased binding with the Bp-R3-ES selected phage indicating that these antigens may be excreted or secreted into the host vasculature. It should be noted that the proteins observed in Table 1 were often identified in one life-stage by proteomic analysis, but had expressed sequence tags (ESTs) in many life-stages or vice versa. This apparent inconsistency may reflect the incompleteness of the proteomic datasets available for analysis or the fact that mRNA levels do not necessarily correlate with protein expression.

### Bp-R3-AES scFv-phage pools recognise adult schistosome protein extracts

We pooled the Bp-R3-A and Bp-R3-ES to widen the pool of potential phage sequences selected for adult and secreted schistosome epitopes and analysed the specificity of the pooled Bp-R3-AES library to bind to different schistosome antigen preparations by ELISA (Fig 2). The Bp-R3-AES phages showed significant levels of binding towards SWAP (p < 0.05) (Fig 2A) indicating they recognised adult proteins. There was no observed binding of the Bp-R3-AES pool to the SEA preparation (Fig 2B) and background binding by M13K07 helper-phage to any antigen preparation was negligible (Fig 2A and 2B). Western blotting of Bp-R3-AES phages was also performed against *S*. *japonicum* derived SWAP and SEA preparations (Fig 2C). The Bp-R3-AES phages bound to a broad range of epitopes within the SWAP preparation, but as observed using ELISA, did not recognise any epitopes within the SEA preparation (Fig 2C; Lane 2). Although the lack of SEA reactivity was unexpected, for a diagnostic aimed to detect an active adult infections, lack of SEA reactivity could be advantageous.

### Isolated soluble scFv clones preferentially recognise SWAP antigens

**Fig 3:**
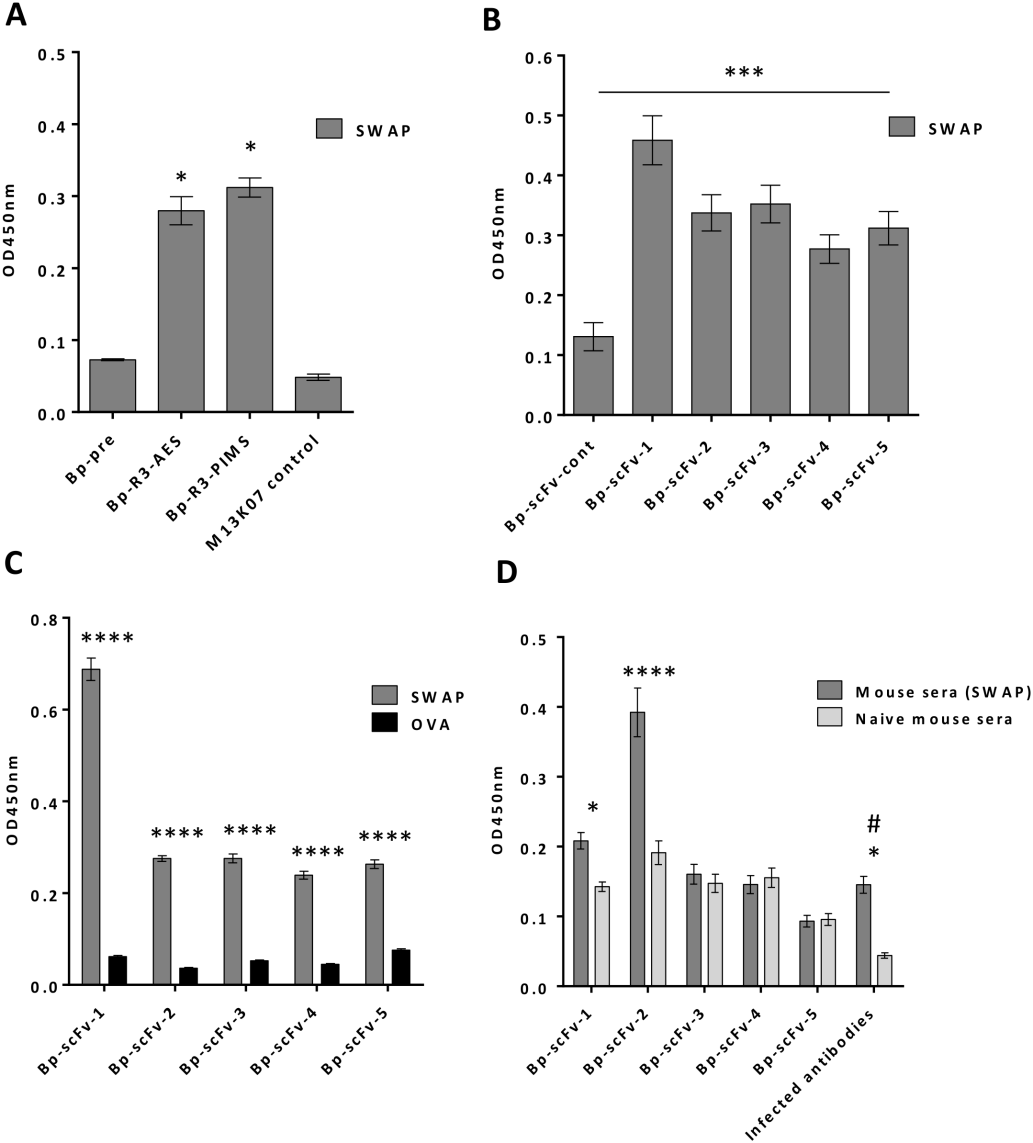
Binding of buffalo portal-round three selected (Bp-R3) single-chain antibody Fv domain (scFv) phage panned against infected mouse sera and scFv-alkaline phosphatase (scFv-AP) fusion proteins to *Schistosoma japonicum* soluble whole adult preparation (SWAP) by ELISA. Data represent mean ± S.E.M. of the O.D._450nm_ for Bp-R3-A and ES pooled phage libraries (Bp-R3-AES) and Bp-R3-post-infected-mouse-sera (Bp-R3-PIMS) phage binding to *S*. *japonicum* SWAP (A), and scFv-AP proteins (Bp-scFv-1 to Bp-scFv-5) binding to SWAP (B and C) or SWAP spiked naive mouse sera (D). Antibodies derived from an *S*. *japonicum* infected rabbit (infected antibodies) were used as a positive control (D#). Statistical significance was determined by one- or two-way ANOVA followed by Tukey’s post-hoc test, where * *P* < 0.05, *** *P* < 0.001, **** *P* < 0.0001.

The buffalo derived Bp-R3-AES phages were further panned against infected mouse sera to eliminate scFv-phage sequences that bind epitopes within sera, and enrich for antigens that are excreted into the serum during an experimental infection. This process generated a library designated Bp-R3-PIMS, which displayed similar binding to SWAP proteins when compared to the Bp-R3-AES libraries as determined by ELISA (Fig 3A). Five soluble alkaline phosphatase (AP) scFv fusion proteins were generated from the Bp-R3-PIMS library (Bp-scFv-1 to Bp-scFv-5; S3 Fig).

All identified scFv-AP proteins (Bp-scFv-1 to Bp-scFv-5) showed significantly higher binding to SWAP relative to an ovalbumin protein binding control as examined by ELISA (Fig 3C). An *E*. *coli* lysate control, containing no expressed scFv-AP protein, showed no reactivity against SWAP protein extract (Fig 3B). Two of the scFv-AP clones (Bp-scFv-1 and Bp-scFv-2) were also able to significantly and preferentially recognise SWAP proteins that had been spiked into naïve mouse sera by ELISA (Fig 3D)

**Fig 4:**
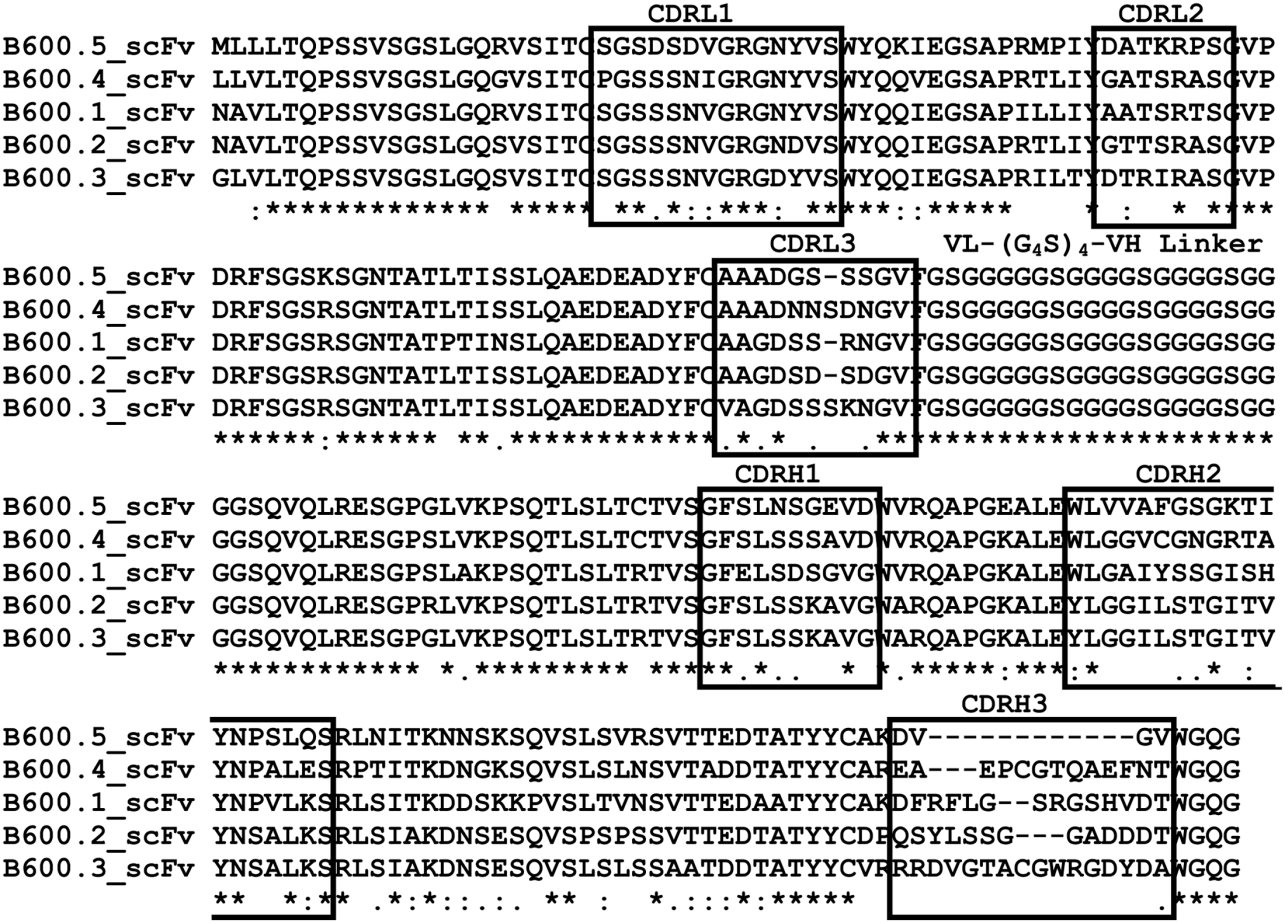
Clustal Omega alignment of soluble single-chain antibody Fv domain-alkaline phosphatase (scFv-AP) antibody sequences. Complementarity determining regions (CDR) regions of variable light (CDRL1-3) and variable heavy (CDRH1-3) chains are indicated in boxes. Respective VL and VH regions show similarity within the framework regions and distinct differences within CDR regions, particularly within CDRH3. (*) indicates a fully conserved residue, (:) a strongly similar and (.) a weakly similar residue.

Sequencing of the scFv-AP clones (Fig 4) revealed high conservation of framework sequences within both the VH and VL regions with significant variations only within the complementarity determining (CDR) regions. As expected within antibody sequences, the major region of diversity observed was within the CDR 3 of the heavy chain (CDRH3; Fig 4) with amino acid sequence length ranging from 4–16 amino acids.

## Discussion

There has been considerable success at reducing transmission and infection rates of *S*. *japonicum* in the PR China [42], however, there is a need to ensure the parasite life cycle is completely broken and that the national elimination program does not falter due to a significant number of low-level infections not being detected [11]. In areas where schistosomiasis japonica remains a problem there is a need to measure and target treatments as well as to educate communities [43]. If a significant number of low-level infections go undetected, there is the risk that the efforts already employed to control transmission will be in vain [11].

The Kato-Katz stool smear technique has been the backbone of intestinal schistosomiasis diagnosis in epidemiological studies, and in the case of *S*. *japonicum* is the only approved measure for diagnosis of current infection [20, 44]. However, the technique is becoming less useful in regions where control programs have resulted in light parasite infections [45, 46]. The vast majority of diagnostic measures for schistosomiasis are underestimating parasite burdens [47, 48]. This is of particular concern for prevention strategies being employed in regions of PR China where accurate diagnosis is crucial for the effective control and surveillance of the disease [49]. The most recent epidemiological surveys suggest that the prevalence of *S*. *japonicum* infection in areas where transmission has not yet been controlled is 5.1% [49] and, as the country strives for elimination of schistosomiasis, the need for more reliable diagnostic tests to survey parasite prevalence is essential [50].

A number of diagnostic tests that detect the host antibody response to schistosome infection have been developed and are being integrated into national control programs in endemic areas of PR China. These include the circumoval precipitin test (COPT), the silver-enhanced colloidal gold metalloimmunoassay, enzyme-linked immunosorbent assay (ELISA), indirect hemagglutination assay (IHA), dot immunogold filtration assay (DIGFA) and dipstick dye immunoassay (DDIA), [44, 51–53]. These tests provide a more accurate measure of infection than stool based parasitological tests with greater patient compliance as they are less invasive [44]. The DDIA test is now commercially available as a test for *S*. *japonicum* in low endemic regions [53]. However, it should be noted that due to the lack of strict approval guidelines in PR China, many poor performing diagnostic tests are also being used [44]. A study by Cai and colleagues further confirmed three of the antibody based diagnostic tests (IHA, ELISA and DDIA) successfully identify schistosomiasis infected patients [49]; however none are able to accurately distinguish between current or cured infection status and thus assessment of treatment outcomes is difficult.

More recently a number of assays to detect circulating antigen have been described. Detection of two circulating diagnostic antigens, circulating cathode antigen (CCA) and circulating anode antigen (CAA), have been developed into diagnostic tests for detection and diagnosis of schistosomiasis. A point-of-care (POC) CCA test is now commercially available and has proven successful for detection of intestinal *S*. *mansoni*, and has been proposed as an alternative to the replicate Kato-Katz measurements [20, 54]. The POC-CCA test is effective in endemic regions, yet its efficacy in areas of low intensity infections requires validation [14, 18, 55, 56]. Recent work by van Dam and colleagues demonstrated that a new technique to enhance CAA detection in sera and urine was six fold more sensitive at diagnosing an active schistosome infection when compared to Kato-Katz measurements [14]. The significant advantage of a sensitive and reliable antigen based diagnostic for schistosome infection is the ability to discriminate active infection. Yet, immunological tests are currently still expensive and often require trained technicians for the administration and analysis, which increases cost and demand for infrastructure [57]. Moreover, the ability of these tests to assess drug efficacy and provide information regarding the impact of control interventions stills needs to be evaluated [20].

The zoonotic nature of *S*. *japonicum* makes animals, particular water buffaloes, significant infection reservoirs. Water buffalo have been thought to contribute to 75% of human infections [13, 58] and prevalence of infection within buffalo populations has been reported to be as high as 10% [59]. It is likely that control within humans living in close proximity to water buffalo will require accurate diagnosis of residual infection within these buffalo populations. This will likely require a low cost easy to administer test that accurately determines infection status.

The novel phage display approach used in this study incorporates the natural reservoir host of *S*. *japonicum* to generate mature antibody fragments that bind to adult schistosomes and ES products. The matured buffalo antibody fragments should show little cross-reactivity with buffalo proteins and may help form the basis of a reagent to diagnose infection in buffalo. Furthermore, we have recently shown that buffalo CDRH3 regions selected for binding to schistosomules using the antibody phage display system are longer than those from non-selected libraries, suggesting that long CDRH3 regions may be important in host parasite antigen recognition [22]. Recent studies have shown that long CDRH3 regions are pivotal in the successful defence against dangerous viral infections such as HIV [60] and although the functional mechanisms for ultra-long CDRH3 regions in cattle has yet to be elucidated [61] they may offer advantages for diagnostic development. Although we did not observe ultra-long CDRH3 regions in the five soluble scFv-AP clones characterised they are only a very small subset of the total number of clones generated.

Previous studies have utilised phage display technology to investigate the maturation of displayed antibody or peptide fragments toward specific *S*. *japonicum* molecules [62–64]. These studies primarily involved the identification of peptide binders through the use of commercial peptide-phage display libraries. The present study used the immunological status of infected host animals to select from a B-cell repertoire primed to produce variable regions that recognise immune exposed epitopes present in the host at the time of sacrifice, i.e. when adult parasites reach the hepatoportal circulation. Previous studies by McWilliam et al., have illustrated the ability of selected activated lymph node antibodies to discriminate against particular stages of *S*. *japonicum* [21, 28]. We have expanded on this principle by producing scFv-phage libraries from activated lymph nodes to allow molecular characterisation and easier production of the binding moity, which can be difficult to isolate from lymph node antibody preparations.

Following selection against defined antigen pools, the binding profile of Bp-R3 phages against SWAP antigens still displayed reactivity to a broad range of antigens, with significant recognition towards three distinct regions, suggesting affinity maturation of Bp-R3 phages towards specific antigens within SWAP. Examination of Bp-R3 phages by ELISA revealed that Bp-R3 phages selected against adult worms or ES preparations were positively and significantly selected towards the antigenic preparations for which they were panned. This observation indicates the maturation of different antibody paratopes within the two scFv-phage libraries. However, it should be noted that microscopy and ELISA observations did indicate cross-reactivity of the differentially selected Bp-R3-A and Bp-R3-ES phage libraries. When analysed on a protein microarray, specific for schistosome proteins, the Bp-R3 phage libraries bound a number of known and hypothetical proteins. Importantly, the library enriched on ES derived material displayed stronger binding to all detected proteins, suggesting that these epitopes may be secreted by the parasite and are presented within the blood of infected individuals. Interestingly, three of the proteins recognised (**AY814158**, **AY815690** and **AY915388**) have previously been observed using an scFv-phage library selected against the larval stages of *S*. *japonicum* [22]. Recent work by Silas and colleagues has identified splice variants of tropomyosin (**AY809972**) within *S*. *mansoni* that may be associated with natural immunity within the definitive host [39]. Their work identified high IgE and IgG4 antibody titres following exposure to *S*. *mansoni* tropomyosin variants indicating tropomyosin is available to the immune system during a natural infection.

This present work also identified and characterised five unique soluble scFv-AP clones selected from the Bp-R3-PIMS phage library, which had been panned against serum obtained from *S*. *japonicum* infected mice. This blood panning, in particular the initial selection against naïve sera, was performed to both eliminate scFvs that preferentially bind to blood proteins and enrich for scFv-phages that bind schistosome antigens present in blood following infection. All of the isolated soluble scFv-AP clones (Bp-scFv-1 to Bp-scFv-5) significantly bound to SWAP proteins. Importantly a lysate control, containing no expressed scFv, did not recognise SWAP proteins and no purified scFv-AP clone specifically recognised ovalbumin, a non-related protein binding control. Only two of the five scFv-AP clones were able to significantly detect SWAP above the level of background mouse blood proteins in an ELISA using naïve mouse sera spiked with SWAP antigens. This suggests that the scFv-phage technique can isolate antibody fragments that recognise schistosome antigens in blood. However, further validation and isolation of these scFv antibody fragments is required to ensure they exclusively recognise schistosome antigens in naturally infected animals.

The development of a range of approaches to deliver accurate point of care, rapid schistosome diagnosis will be critical in the fight to eradicate schistosomiasis japonica in areas of low but continuing transmission in PR China. Here we describe the use of the natural buffalo host to develop and identify recombinant scFv fragments that bind adult stage and ES antigens from *S*. *japonicum*. The isolated soluble scFv clones require further development, purification and testing within a true schistosomiasis japonica infection setting, but once completed could be valuable reagents in the construction of a cost effective point-of-care diagnostic test.

## Supporting Information

S1 Fig Cloning procedure and amplification of single-chain antibody Fv domain (scFv) inserts. Total RNA from the portal-LN collected 11–12 days post *Schistosoma japonicum* cercarial infection was reverse transcribed to cDNA. Variable heavy (VH) and variable light (VL) genes were amplified (A). Full length scFv fragments were assembled by splice overlap PCR and the full length scFv construct was amplified using scFv specific primers (A#). Molecular weights in base-pairs (bp) are indicated. Full length scFv fragments were inserted into pAK100 vector via *Sfi*I cloning. Selected phagemid were sequenced and aligned to cattle variable regions (B). Shaded regions represent complementarity determining regions (CDR) for VH and VL genes; (*) indicates a fully conserved residue, (:) a strongly similar and (.) a weakly similar residue.
(TIF)

S2 Fig Relative enrichment of functional single-chain Fv domain (scFv) phage particles. Binding to adult *Schistosoma japonicum* worms and excretory secretory (ES) products was determined using the relationship between the output titre from scFv-phage eluted from adult *S*. *japonicum* worms or ES products at each round and control reactions containing no parasite material.
(TIF)

S3 Fig Expression of soluble single-chain Fv domain (scFv) alkaline phosphatase (AP) fusion proteins. Soluble scFv-AP clones were expressed and probed with anti-AP-HRP and positive expression is indicated. A soluble scFv-His tag control protein was expressed and probed with anti-His-HRP and is also indicated. Molecular weights in kilodaltons (kDa) are indicated on the left hand side.
(TIF)
