# Supplementary figures and images for: Generation of a Novel Bacteriophage Library Displaying scFv Antibody Fragments from the Natural Buffalo Host to Identify Antigens from Adult Schistosoma japonicum for Diagnostic Development
Source: PLoS Negl Trop Dis. 2015 Dec 18;9(12):e0004280. doi: 10.1371/journal.pntd.0004280 (PMC4686158; doi:10.1371/journal.pntd.0004280)

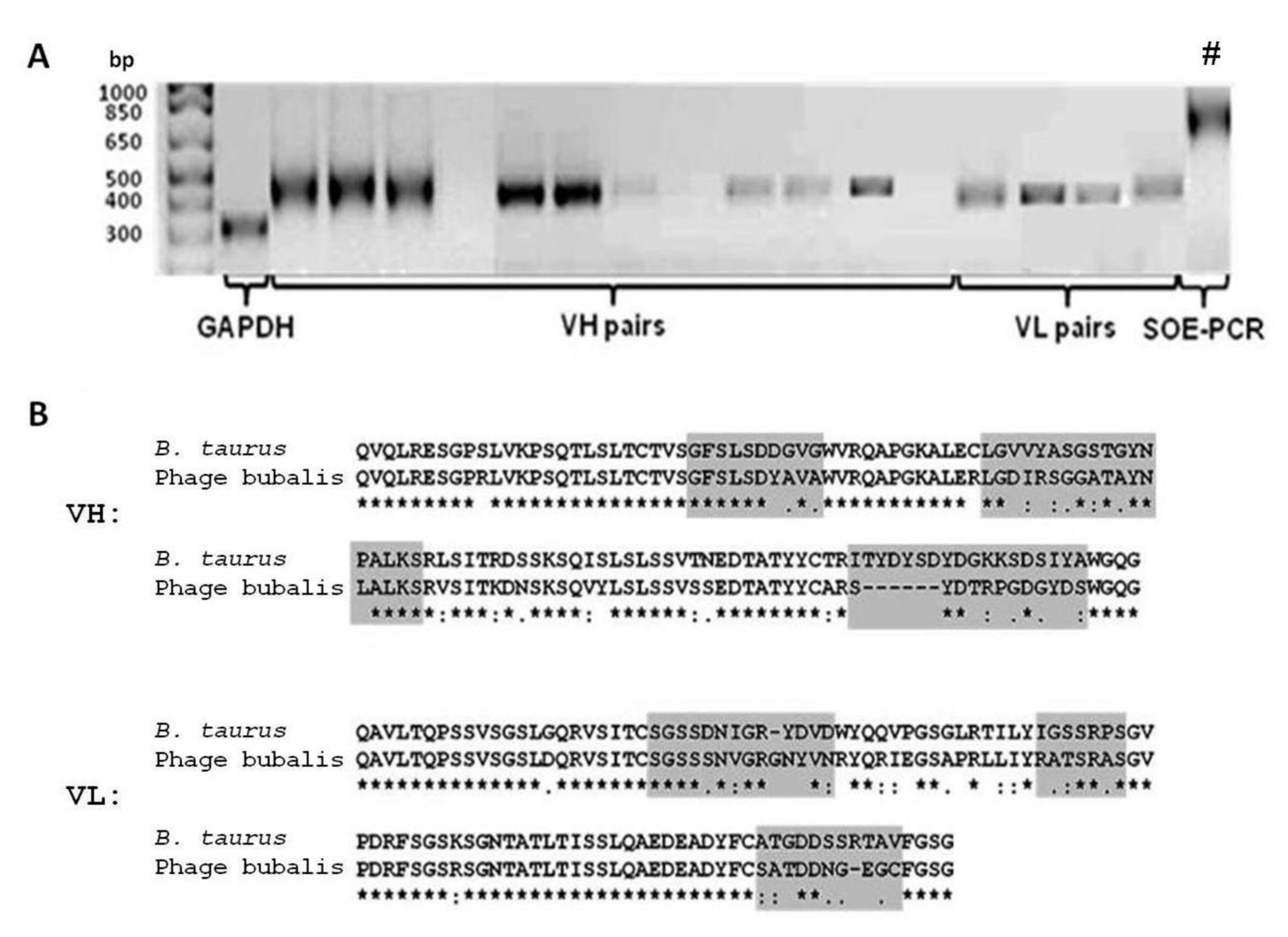

Supplement: S1 Fig — Total RNA from the portal-LN collected 11–12 days post Schistosoma japonicum cercarial infection was reverse transcribed to cDNA. Variable heavy (VH) and variable light (VL) genes were amplified (A). Full length scFv fragments were assembled by splice overlap PCR and the full length scFv construct was amplified using scFv specific primers (A#). Molecular weights in base-pairs (bp) are indicated. Full length scFv fragments were inserted into pAK100 vector via SfiI cloning. Selected phagemid were sequenced and aligned to cattle variable regions (B). Shaded regions represent complementarity determining regions (CDR) for VH and VL genes; (*) indicates a fully conserved residue, (:) a strongly similar and (.) a weakly similar residue. (TIF) [file pntd.0004280.s001.tif]

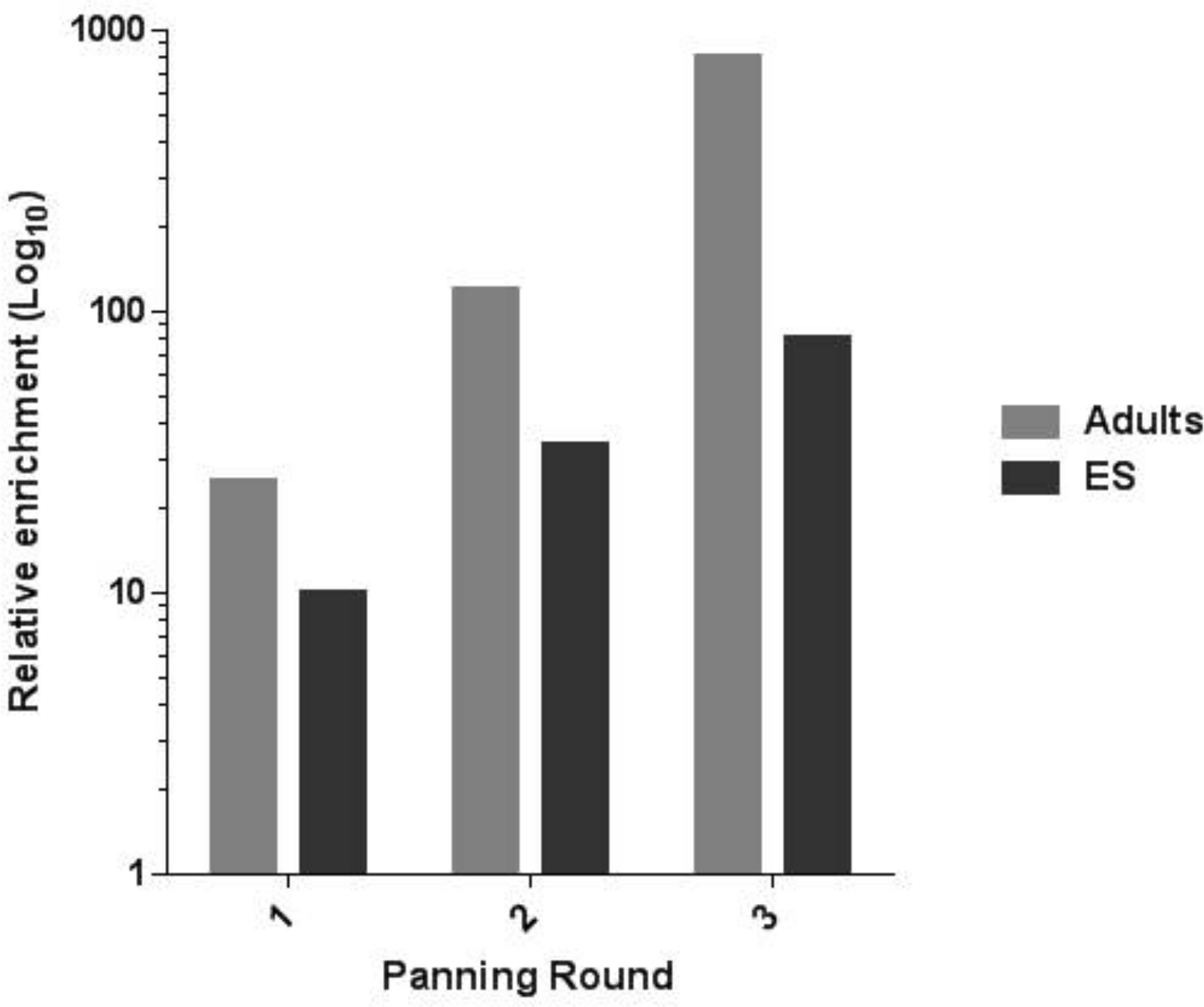

Supplement: S2 Fig — Binding to adult Schistosoma japonicum worms and excretory secretory (ES) products was determined using the relationship between the output titre from scFv-phage eluted from adult S. japonicum worms or ES products at each round and control reactions containing no parasite material. (TIF) [file pntd.0004280.s002.tif]

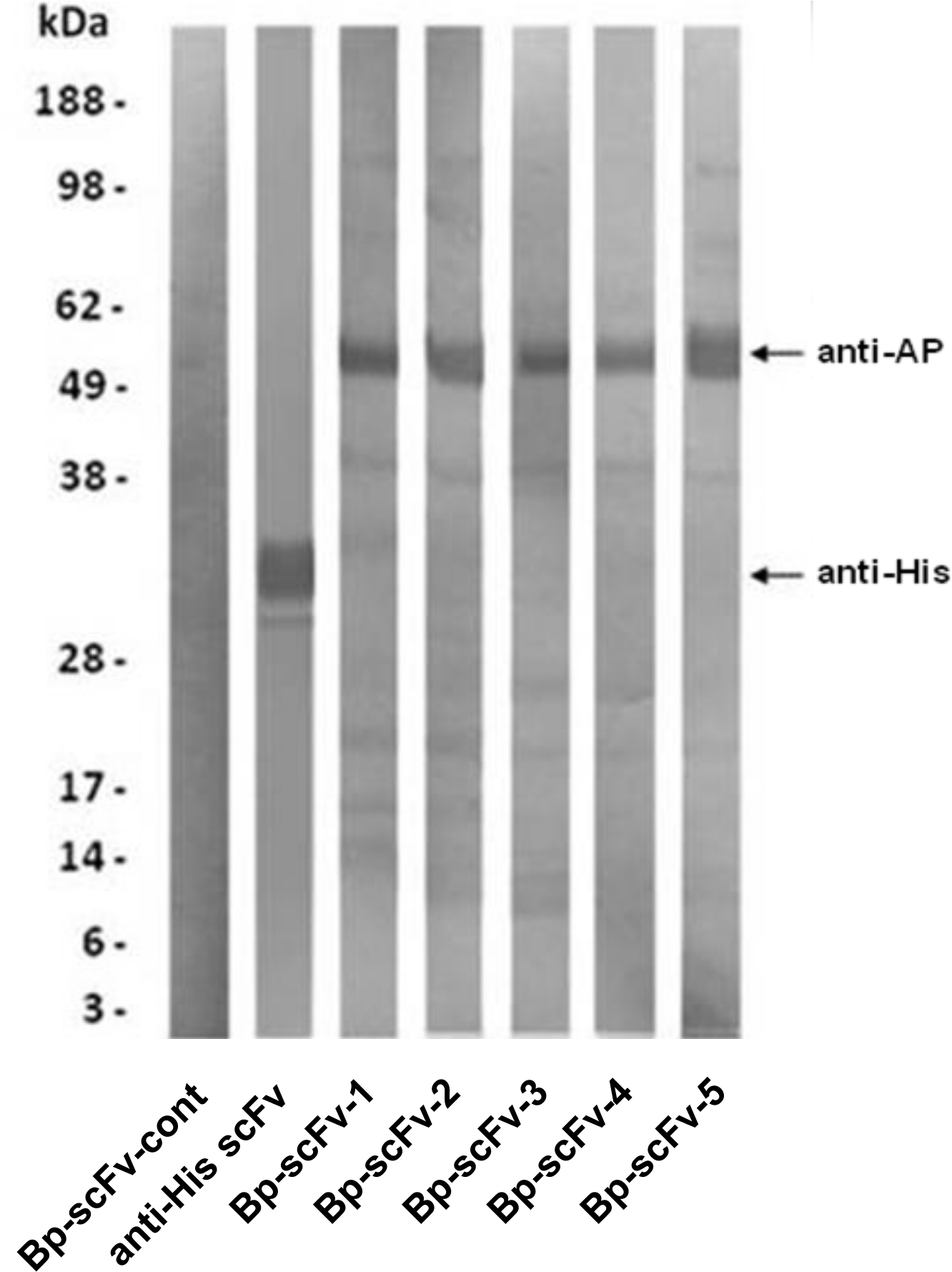

Supplement: S3 Fig — Soluble scFv-AP clones were expressed and probed with anti-AP-HRP and positive expression is indicated. A soluble scFv-His tag control protein was expressed and probed with anti-His-HRP and is also indicated. Molecular weights in kilodaltons (kDa) are indicated on the left hand side. (TIF) [file pntd.0004280.s003.tif]
